# The impact of allergic rhinitis on functional, aesthetic and scar outcomes after open septorhinoplasty: a prospective controlled study

**DOI:** 10.1017/S0022215126104800

**Published:** 2026-06

**Authors:** Tankut Uzun, Eray Uzunoğlu

**Affiliations:** Department of Otolaryngology and Head & Neck Surgery, Cigli Training and Research Hospital, Bakircay University Faculty of Medicine, Izmir, Turkey

**Keywords:** rhinitis, allergic, rhinoplasty, nasal obstruction, wound healing, patient satisfaction

## Abstract

**Objective:**

To compare 12-month functional, aesthetic and scar outcomes after septorhinoplasty in patients with and without allergic rhinitis and to assess allergy symptoms in the allergic rhinitis group.

**Methods:**

Ninety-six patients were included (allergic rhinitis, *n* = 47; non-allergic rhinitis, *n* = 49). No turbinate surgery was performed. Allergy symptoms (allergic rhinitis group) were rated pre-operatively and at 12 months using a visual analogue scale (VAS). At 12 months, all patients completed the Standardised Cosmesis and Health Nasal Outcomes Survey; scars were evaluated with the Stony Brook Scar Evaluation Scale and patient- and physician-reported VAS scores for scar and nasal shape.

**Results:**

The Standardised Cosmesis and Health Nasal Outcomes Survey, the Stony Brook Scar Evaluation Scale and scar VAS scores were similar between groups. Nasal shape VAS scores were higher in the non-allergic rhinitis group, significant only for physician ratings (*p* = 0.03). In the allergic rhinitis group, allergy VAS decreased from 39.32 ± 42.25 to 24.22 ± 31.96 (*p* < 0.001).

**Conclusion:**

Allergic rhinitis does not adversely affect functional, aesthetic or scar outcomes after septorhinoplasty, and septorhinoplasty may clinically reduce allergy symptoms in patients with allergic rhinitis.

## Introduction

Allergic rhinitis is an inflammatory disorder of the nasal mucosa characterised by nasal congestion, rhinorrhoea, sneezing and nasal itching, all of which can negatively affect respiratory function.^1^ These symptoms frequently coexist with structural abnormalities such as septal deviation, and substantially diminish patients’ quality of life. As a chronic inflammatory condition, allergic rhinitis has the potential to influence the outcomes of various respiratory and nasal surgical procedures, prompting numerous investigations into its impact on post-operative results.^2^^–^^4^

There is substantial evidence that both septorhinoplasty and septoplasty lead to improvements in quality of life and reductions in allergy-related symptoms among patients with allergic rhinitis.^2^^,^^3^ Moreover, several studies have demonstrated significant post-operative improvements in both allergic and non-allergic individuals, indicating that nasal surgery can be safely performed in the presence of allergic rhinitis and can yield meaningful functional benefits regardless of underlying allergic status.^4^^,^^5^

While the limited number of studies in the literature have primarily focused on aesthetic outcomes or overall symptom improvement following septorhinoplasty, few have offered a comprehensive evaluation that simultaneously examines scar healing, nasal function, aesthetic satisfaction and changes in allergy-related symptoms. Addressing this gap, our prospective controlled trial was designed to compare allergic and non-allergic patients across all of these domains, with the aim of providing holistic and objective data on how underlying allergic status may influence post-operative recovery, symptom trajectories and patient-reported outcomes. We believe that this multidimensional and comparative approach will offer a more complete understanding of septorhinoplasty outcomes than what is currently available in the literature and will contribute meaningful new perspectives to clinical practice.

## Material and methods

This study was designed as a prospective controlled clinical study comparing patients with and without allergic rhinitis who underwent septorhinoplasty. The study protocol was approved by the Institutional Ethics Committee of Izmir Bakircay University. Written informed consent was obtained from all participants prior to enrolment. All procedures adhered to the principles of the Declaration of Helsinki.

The study included patients aged 18–50 years who underwent septorhinoplasty in our clinic between November 2023 and November 2024. Individuals with a history of nasal trauma, prior nasal surgery, systemic steroid use, autoimmune disorders, chronic inflammatory or rheumatological diseases, or active sinonasal infection were excluded. Smokers and patients undergoing revision surgery were also excluded.

Participants were allocated into two groups according to the presence or absence of allergic rhinitis. The diagnosis of allergic rhinitis was based on clinical history, including persistent symptoms of nasal itching, congestion, sneezing and rhinorrhoea, together with the use of intranasal steroids or antihistamines within the previous year. The allergic rhinitis group consisted of patients whose diagnosis had been confirmed by a specialist physician, who demonstrated elevated immunoglobulin E levels on laboratory testing and who had been followed with this diagnosis for at least one year. Patients without these symptoms and without a history of anti-allergic medication and allergic symptoms served as the non-allergic rhinitis control group.

The sample size was determined *a priori* based on between-group comparison of post-operative patient-reported nasal appearance visual analogue scale (VAS) scores (primary outcome). With *α* = 0.05, 1 − *β* = 0.80 and an assumed moderate effect size (Cohen’s *d* = 0.6), a minimum of 46 patients per group was required. The same sample size also provides adequate power to detect moderate between-group differences in other continuous outcomes (e.g. Standardised Cosmesis and Health Nasal Outcomes Survey total scores), whereas small effect sizes cannot be reliably excluded.

The study initially enrolled 50 patients in each group to account for potential loss to follow up. During the follow-up period, three patients in the allergic rhinitis group and one patient in the non-allergic group were excluded because of failure to attend scheduled post-operative assessments. Thus, the study was completed with a total of 96 patients who had both pre-operative and 1-year post-operative data available for analysis. All patients were followed for 12 months(1-, 3-, 6- and 12-month routine controls).

### Surgical technique

All surgical procedures were performed by the same senior surgeon using an open septorhinoplasty approach to ensure technical consistency. Standardised operative steps were applied, including dorsal septal reconstruction, tip support manoeuvres and correction of internal and external nasal valve dysfunctions as needed. Inferior turbinate procedures (radiofrequency ablation or partial turbinoplasty) were not performed. No additional mucosal procedures related to allergic rhinitis were performed.

### Outcome measures

#### Allergy Symptom Assessment (visual analogue scale)

Allergic symptoms—including nasal obstruction, rhinorrhoea, sneezing and nasal itching—were evaluated in patients with allergic rhinitis using separate 100-mm VASs. Scores ranged from 0 (no symptoms) to 100 (severe symptoms). VAS measurements were recorded pre-operatively and at the one-year post-operative follow up.

#### Functional and aesthetic outcomes (Standardised Cosmesis and Health Nasal Outcomes Survey)

At 12 months post-operatively, all patients completed the Standardised Cosmesis and Health Nasal Outcomes Survey^6^ (Table 1). This survey was administered under standardised conditions by a researcher blinded to the allergic rhinitis status of the patients.

**Table 1.** Standardised Cosmesis and Health Nasal Outcomes Survey. Over the past month, how much of a problem was the following? Please rate each item on a scale from 0 (no problem) to 5 (extreme problem) Table 1 long description.

Item	0	1	2	3	4	5
1 Having a blocked or obstructed nose						
2 Getting air through my nose during exercise						
3 Having a congested nose						
4 Breathing through my nose during sleep						
5 Decreased mood and self-esteem due to my nose						
6 The shape of my nasal tip						
7 The straightness of my nose						
8 The shape of my nose from the side						
9 How well my nose suits my face						
10 The overall symmetry of my nose						

#### Scar healing assessment (Stony Brook Scar Evaluation Scale)

Objective scar appearance was evaluated using the Stony Brook Scar Evaluation Scale, adapted for columellar scar assessment, at the one-year follow up^7^ (Table 2). The scale assesses scar width, elevation and/or depression, discoloration, notching and overall appearance. Scoring was performed by an independent physician who was blinded to group allocation and clinical outcomes.

**Table 2.** Stony Brook Scar Evaluation Scale (adapted for columellar scar) Table 2 long description.

Scar category	Criteria	Points
Width	>2 mm/≤2 mm	0/1
Height	Depressed or elevated from surrounding skin/flat	0/1
Discoloration	Darker than surrounding skin/same colour or lighter	0/1
Notching	Present/absent	0/1
Overall appearance	Poor/good	0/1

#### Independent aesthetic visual analogue scale evaluation

Postoperative nasal aesthetic appearance (nasal shape) was independently assessed by a blinded physician and the patients using a 100-mm visual analogue scale (VAS; 0 = unacceptable appearance, 100 = excellent appearance) at the one-year follow-up.

## Results

A total of 96 patients who underwent septorhinoplasty were included in the study. The mean age of the cohort was 26.93 ± 7.42 years (range, 18–48 years). Of the participants, 69 were female (71.9 per cent) and 27 were male (28.1 per cent). Based on clinical history, 47 patients (49.0 per cent) were classified as having allergic rhinitis, while 49 patients (51.0 per cent) had no history of allergic disease. There were 33 female and 14 male patients in the allergic rhinitis group, and 35 female and 14 male patients in the non-allergic group. The mean age was 27.36 ± 7.28 years in the allergic rhinitis group and 26.51 ± 7.61 years in the non-allergic group. The demographic characteristics, including age and sex distribution, were comparable between the two groups.

The Standardised Cosmesis and Health Nasal Outcomes Survey and the Stony Brook Scar Evaluation Scale results at one-year follow up are presented in Tables 3 and 4. No statistically significant differences were observed between patients with and without allergic rhinitis in any of the aesthetic, functional or wound-healing parameters assessed by these two instruments (*p* > 0.05). The VAS scores evaluated by the patients and by the independent blinded physician are shown in Table 5. Both patient- and physician-reported VAS scar scores were comparable between the groups (*p* > 0.05).

**Table 3.** Comparison of Standardised Cosmesis and Health Nasal Outcomes Survey scores between allergic and non-allergic patients Table 3 long description.

Parameter	Allergic patients (mean ± SD)	Non-allergic patients (mean ± SD)	*p* value
1	1.38 ± 1.38	1.29 ± 1.29	0.743
2	1.00 ± 1.18	0.88 ± 1.20	0.534
3	1.62 ± 1.41	1.18 ± 1.22	0.149
4	1.09 ± 1.36	1.12 ± 1.48	0.921
5	0.53 ± 1.14	0.47 ± 0.96	0.980
6	0.85 ± 1.22	0.84 ± 1.39	0.653
7	0.62 ± 0.95	0.63 ± 1.09	0.677
8	0.49 ± 0.98	0.45 ± 0.96	0.545
9	0.36 ± 0.64	0.39 ± 0.91	0.523
10	0.87 ± 1.19	0.76 ± 1.25	0.465
Total	8.83 ± 7.84	7.86 ± 7.57	0.386

SD = standard deviation

**Table 4.** Comparison of the Stony Brook Scar Evaluation Scale scores between allergic and non-allergic patients Table 4 long description.

Parameter	Allergic patients (mean ± SD)	Non-allergic patients (mean ± SD)	*p* value
Width*	1	1	1
Height	0.87 ± 0.34	0.86 ± 0.35	0.829
Discoloration	0.96 ± 0.20	0.98 ± 0.14	0.535
Notching	0.89 ± 0.31	0.92 ± 0.28	0.679
Overall appearance	0.98 ± 0.15	1.00 ± 0.00	0.307
Total	4.74 ± 0.71	4.86 ± 0.41	0.813

* All patients received a score of 1. SD = standard deviation

**Table 5.** Comparison of patient- and physician-reported VAS scores between allergic and non-allergic groups Table 5 long description.

Parameter	Allergic patients (mean ± SD)	Non-allergic patients (mean ± SD)	*p* value
VAS scar			
– Patient	94.11 ± 9.55	94.18 ± 8.38	0.913
– Physician	94.04 ± 6.89	94.90 ± 5.54	0.735
VAS nasal shape			
– Patient	88.62 ± 10.82	91.94 ± 9.99	0.080
– Physician	88.83 ± 4.06	91.12 ± 5.52	0.033

SD = standard deviation

For the VAS ratings assessing satisfaction with nasal shape, higher scores were noted in the non-allergic group for both patient-reported (*p* = 0.08) and physician-reported (*p* = 0.03) evaluations; however, the difference reached statistical significance only in the physician-reported VAS score. Among patients with allergic rhinitis, a statistically significant reduction was observed between pre-operative (39.32 ± 42.25) and post-operative (24.22 ± 31.96) allergy-related VAS scores (*p* < 0.001). In the allergic rhinitis group, the reduction in allergy-related VAS scores from pre-operative to post-operative assessments showed a large effect size (Cohen’s *d* = 0.88).

### Statistical analyses

All statistical analyses were performed using SPSS Statistics version 20.0 (IBM Corp., Armonk, NY, USA). The normality of continuous variables was assessed using Kolmogorov–Smirnov and Shapiro–Wilk tests. Descriptive statistics were presented as mean ± standard deviation for continuous variables and as frequencies and percentages for categorical variables.

Comparisons between allergic rhinitis and non-allergic groups were conducted using the Mann–Whitney U test for non-normally distributed continuous variables, including Standardised Cosmesis and Health Nasal Outcomes Survey scores, Stony Brook Scar Evaluation Scale scores, and patient-reported and physician-reported VAS scar scores and VAS nasal appearance scores. For patients with allergic rhinitis, pre-operative and post-operative allergy-related VAS scores were compared using the Wilcoxon signed-rank test, given the non-normal distribution of repeated measurements. A 2-tailed *p* value less than 0.05 was considered statistically significant.

## Discussion

In septorhinoplasty—a procedure in which patient satisfaction is a primary outcome—aesthetic results represent the strongest determinant of overall satisfaction, yet functional improvement also plays a meaningful and clinically significant role in shaping patient-reported outcomes.^8^ While many parameters influence outcomes in septorhinoplasty patients, the effect of allergic rhinitis, which is one of the potential contributing factors, has unfortunately been explored in only a limited number of studies.

Studies have shown that patients with allergic rhinitis who undergo septorhinoplasty typically experience a reduction in allergy-related symptoms and an improvement in respiratory function; however, these studies have not evaluated important outcomes such as wound healing or satisfaction with aesthetic results.^3^^,^^4^^,^^9^

Unlike previous studies, which have focused primarily on functional improvement or allergy-related symptom changes, our study is original in that it provides a multidimensional assessment encompassing functional outcomes, aesthetic satisfaction and post-operative wound healing. In our study, no statistically significant differences were found in any of the items of the Standardised Cosmesis and Health Nasal Outcomes Survey questionnaire, which assesses functional and aesthetic outcomes collectively, between patients with and without allergic rhinitis; both groups demonstrated comparable levels of post-operative satisfaction. Similar to our results, Sokoya *et al*.^4^ reported that patients with and without allergic rhinitis experienced comparable improvements in The NOSE (Nasal Obstruction Symptom Evaluation) scale scores after functional open septorhinoplasty, concluding that allergic rhinitis does not adversely affect functional outcomes and that open septorhinoplasty may even offset the negative effects of allergic inflammation on nasal obstruction.

Gillman *et al*.^5^ reported that inferior turbinate surgery performed in combination with septoplasty leads to a reduction in allergic rhinitis symptoms. There are meta-analyses and systematic reviews in the literature demonstrating that turbinate surgical procedures lead to a significant reduction in allergic rhinitis symptoms.^10^^,^^11^ In contrast, our study evaluated the effects of isolated septorhinoplasty without concomitant turbinate surgery and demonstrated a statistically significant improvement between pre-operative and post-operative VAS scores in patients with allergic rhinitis. This finding is particularly noteworthy because it indicates that septorhinoplasty alone may also confer meaningful symptomatic benefit in the management of allergic rhinitis. In line with our findings, García-Paz *et al*.^3^ demonstrated that septorhinoplasty in patients with allergic rhinitis and septal deviation resulted in reduced obstruction severity and decreased medication use, as evidenced by improvements in VAS and Cuestionario Español de Calidad de Vida en Rinitis questionnaire scores.

In both groups, patients reported high VAS satisfaction scores for nasal shape, and although scores were slightly higher in the non-allergic group, the difference did not reach statistical significance. In contrast, physician-reported VAS nose shape scores favoured the non-allergic group and demonstrated a statistically significant difference (*p* = 0.03). Similar discrepancies between patient and physician perceptions have been described in the aesthetic surgery literature and may reflect the physician’s heightened sensitivity to subtle oedema, contour irregularities or residual asymmetries that may persist longer in individuals with a tendency toward mucosal inflammation. Nevertheless, the absence of any patient-reported difference in both VAS assessments and the aesthetic items of the Standardised Cosmesis and Health Nasal Outcomes Survey questionnaire suggests that this divergence observed in physician scoring may not translate into a clinically meaningful difference from the patient’s perspective.

The literature on the relationship between allergic rhinitis and post-operative wound healing is limited. A notable strength of our study is the objective evaluation of external wound healing using the Stony Brook Scar Evaluation Scale. Although chronic inflammatory conditions are theoretically expected to impair wound healing, our findings showed no significant differences between groups in any parameter of the scale. Consistent with these findings, both patient- and physician-reported VAS scar assessment scores were also similar between the two groups. This suggests that systemic or localised allergic inflammatory activity does not meaningfully influence external scar maturation following open septorhinoplasty.

- Allergic rhinitis commonly coexists with septal deviation and can worsen nasal obstruction and quality of life
- Septorhinoplasty can improve nasal function and may reduce allergy-related symptoms in patients with allergic rhinitis
- Evidence on whether allergic rhinitis compromises aesthetic satisfaction and, especially, external scar healing after open septorhinoplasty is limited
- In a prospective controlled cohort (allergic rhinitis versus non-allergic rhinitis) undergoing open septorhinoplasty without turbinate surgery, 12-month functional (Standardised Cosmesis and Health Nasal Outcomes Survey), aesthetic and scar (Stony Brook Scar Evaluation Scale and visual analogue scale (VAS) scar rating) outcomes were comparable between groups
- Physician-rated nasal shape VAS was slightly higher in the non-allergic rhinitis group, while patient-rated aesthetic satisfaction did not differ meaningfully
- Allergy symptom burden (VAS) significantly improved at 12 months in the allergic rhinitis group, suggesting septorhinoplasty alone may provide clinical symptom benefit in appropriately selected allergic rhinitis patients

### Limitations

Firstly, this study was conducted at a single centre with a relatively modest sample size and a predominantly young, female population, which may limit the generalisability of the findings to other age groups, sexes or clinical settings. Secondly, patients’ anti-allergic medication regimens were not standardised during the study period, and potential differences in medical management may have influenced the course of allergy-related symptoms. Thirdly, only patient-reported outcome measures and clinical rating scales (the Standardised Cosmesis and Health Nasal Outcomes Survey, VASs and the Stony Brook Scar Evaluation Scale) were used, without complementary objective assessments such as rhinomanometry or acoustic rhinometry.

## Conclusion

Our findings suggest that allergic rhinitis is not a negative prognostic factor for wound healing, functional outcomes or aesthetic results in septorhinoplasty patients. Moreover, in appropriately selected individuals with allergic rhinitis, septorhinoplasty may be considered an important therapeutic option because it is associated with a reduction in allergy-related symptoms.

## Table 1 Long description

The table measures the severity of nasal problems and appearance concerns over the past month, rated from 0 (no problem) to 5 (extreme problem). It includes items such as nasal obstruction, breathing difficulties during exercise and sleep, mood impacts, and nasal aesthetics like shape and symmetry. However, the table lacks specific data points, making it impossible to identify trends or compare the severity of different issues. The absence of responses suggests either a lack of data collection or a need for further input to draw meaningful conclusions.

Navigate back to Table 1.

## Table 2 Long description

The table evaluates columellar scars using the Stony Brook Scar Evaluation Scale, assigning points based on five criteria: width, height, discoloration, notching, and overall appearance. Width is scored 0 if greater than 2 mm and 1 if 2 mm or less. Height is scored 0 if the scar is depressed or elevated and 1 if flat. Discoloration receives 0 points if darker than surrounding skin and 1 if the same color or lighter. Notching is scored 0 if present and 1 if absent. Overall appearance is rated 0 for poor and 1 for good. Each criterion contributes equally to the total score, allowing for a maximum of 5 points, indicating a better scar outcome.

Navigate back to Table 2.

## Table 3 Long description

The table compares standardized cosmesis and health nasal outcomes survey scores between allergic and non-allergic patients. Allergic patients consistently show higher mean scores across most parameters, with parameter 3 showing the largest difference (1.62 vs. 1.18). However, none of the differences between allergic and non-allergic patients are statistically significant, as all p values exceed 0.05. The total mean score for allergic patients is 8.83, slightly higher than the 7.86 for non-allergic patients, but this difference is also not significant (p = 0.386). The data suggests no meaningful statistical difference in outcomes between the two groups.

Navigate back to Table 3.

## Table 4 Long description

The table compares the Stony Brook Scar Evaluation Scale scores between allergic and non-allergic patients across several parameters. Both groups received a score of 1 for width. The mean height score was 0.87 for allergic patients and 0.86 for non-allergic patients, with a p value of 0.829, indicating no significant difference. Discoloration scores were 0.96 for allergic and 0.98 for non-allergic patients, with a p value of 0.535. Notching scores were 0.89 for allergic and 0.92 for non-allergic patients, with a p value of 0.679. Overall appearance scores were 0.98 for allergic and 1.00 for non-allergic patients, with a p value of 0.307. The total scores were 4.74 for allergic and 4.86 for non-allergic patients, with a p value of 0.813. None of the differences between the groups were statistically significant, suggesting similar scar characteristics regardless of allergy status.

Navigate back to Table 4.

## Table 5 Long description

The table compares VAS scores for scar and nasal shape between allergic and non-allergic patients, as reported by both patients and physicians. For VAS scar, both groups have similar scores with no significant difference (p=0.913 for patients, p=0.735 for physicians). However, for VAS nasal shape, non-allergic patients scored higher than allergic patients, with a significant difference in physician-reported scores (p=0.033). Patient-reported scores for nasal shape also show a higher mean for non-allergic patients, but the difference is not statistically significant (p=0.080). These findings suggest that physician assessments of nasal shape may differ more between groups than patient assessments.

Navigate back to Table 5.
